# Treatment of Spermatorrhœa

**Published:** 1872-05

**Authors:** 


*Treatment of Spermatorrhœa.*

The occasional introduction of a catheter as large as the urethra will take, is often of the greatest service; it should be passed into the bladder and allowed to remain for five or ten minutes, according to the tolerance of the patient; its mechanical pressure helps to unload the congested capillaries and small vessels of the urethra; its contact deadens and destroys the extreme sensibility of the urethral nerves, and renders them less susceptible to the influence of slight excitants; whilst, by stimulating the muscles, it provokes their contraction, and so renders material assistance in emptying the larger veins. A silver catheter is the best instrument for the purpose, as it exerts firmer pressure than an elastic bougie; and, as the urine can be drawn off through it, the patient will not require to micturate for several hours, which is a point of some importance, as the urethra is often very tender after the passage of an instrument for the first few times. The frequency with which it should be employed, depends upon the amount of discomfort its presence occasions; and, if the pain be great, it should not be left in more than a few seconds, lest rigors, swelled testicle, etc., be occasioned. Sometimes the urethra is *extremely* sensitive, and much pain attends the use of the catheter; but this is an additional reason for persisting with it, though a smaller one may be employed at first so as to cause less pain. I have sometimes found that smearing the catheter with blue or calomel ointment, or with half a grain to a grain of nitrate of silver rubbed down in an ounce of lard, to be of use in obstinate cases; but I prefer the blue ointment to any thing I have yet tried. Some camphor, extract of opium, belladonna, etc., may be combined with these ointments, if thought desirable. Care should be taken that these applications do not reach much beyond the curve of the instrument, and it should be thoroughly oiled before using it. The oversecretion of mucus is always checked by the use of the catheter, whether armed with ointment or not.

Cold bathing, cold douches, etc., should not be employed on going to bed. The ordinary bath in the morning does good; but cold applications at night should be forbidden, as the reaction which follows them will increase the local circulation, and so cause congestion and erection of the penis, and thus increase the probability of emissions.

Not only must the position assumed in sleep be attended to, but undue warmth in bed avoided, whether by using very soft beds or too large an amount of clothing. The bowels should be carefully regulated, to prevent any accumulation within the rectum; and the urine examined from time to time, so as to detect an excess of uric acid, the presence of oxalates, etc., which may render its passage irritating to the hypersensitive urethra. Overdistension of the bladder must, at all times, be guarded against, and the patient warned to pass urine on waking in the morning, lest he doze off again with a full bladder, which is one of the most certain provocations of erection and emissions.

Before commencing to treat this affection constitutionally, it is generally necessary to allay the digestive disturbances, which are so common and often so severe, by giving such remedies as may be applicable to the condition of the patient, either with or without the more special medicines. By neglecting to do so, we may not only add to the dyspeptic troubles and obtain no benefit from the drugs given, but a valuable medicine may do harm and be brought into disrepute, in consequence of its being administered at a time when the stomach cannot tolerate it.

Internally, I have found astringents of more use in this disorder than tonics; or they may be combined. Gallic acid, the dilute mineral acids, especially the sulphuric, may be given. Tincture of matico will often be of service, and more so, in my experience, than any other plant rich in tannin, as it appears to act upon the genito-urinary tract, rather than upon the bowels, as is often the case with the others.

Ergot is one of the most valuable remedies for this affection, and the liquid extracts of the *Pharmacopœia* is a very efficient and convenient form for giving it; whilst the dilute sulphuric acid can be added, if thought advisable.

When the urethra is very sensitive, and the passage of urine painful, small doses of copaiba are often most comforting; or the other oleoresins may be tried if it disagree; but none of them, in my opinion, is equal in value to copaiba when it can be borne.

I am not disposed to regard strychnine in these cases with very great favor; when there is much irritability of the nerves, I believe it often adds to this; but when this is subsiding it may be of use as a tonic; so may quinine or iron, but in no other way. I have never given the tincture of iron in the enormous doses (from one to two drachms three times daily) recommended by some, and so I cannot speak personally of its value in such large quantity.

Cantharides, phosphorus (except the dilute phosphoric acid), and the so-called aphrodisiacs, do harm by acting as stimulants to the nervous system generally, and therefore to the local nerves. Cantharides, also, by its action upon the bladder, is, especially when given in large doses, a very injurious drug in these cases. For the same reasons I disapprove of local blistering; while the sore left by the blister acts, moreover, as a source of irritation, and adds to the liability of emissions.

Belladonna, in my hands, has proved to be an uncertain remedy; in some cases it has appeared to do good by allaying irritation, whilst in others there were no beneficial results from it. The dryness of the throat, disturbance of vision and diarrhœa, which are often caused by it, constitute an objection to its employment in full doses, and without them its value is very questionable.

Camphor is a most useful drug; three or four grains made up into two pills, with half a grain or a grain of opium and one or two of aloes, have more frequently allayed irritability and prevented emissions, than anything I have yet tried. Opium alone does not succeed as well, and a larger dose is necessary, so that the untoward symptoms sometimes produced by it are more likely to be incurred.

I have tried chloral in a few cases, and with very great advantage: in doses of fifteen or twenty grains at bed-time, it has answered its purpose admirably.

Bromide of potassium, in thirty or forty-grain doses, will sometimes be of service; but it seems to me a less certain remedy than chloral, which I am disposed to regard as one of the valuable agents we possess for these cases, though as yet my experience of it is limited.

Suppositories vary much in their action whatever drugs they may contain. Occasionally they answer well, but often they do not lessen, and I am not sure they do not sometimes increase, the irritability of the parts.

Galvanism I have not employed myself; but in the few instances where I have known of its being tried by others, it has seemed to me to do more harm than good, by adding to the nervous irritation.

Lastly, as to cauterization by the *porte-caustique*, I need scarcely say that I am strongly opposed to this method of treatment; for, if my view of this disorder be correct, this instrument can relieve it in no other way than as the passage of the catheter does. I do not believe that ulceration or other morbid conditions of the ejaculatory ducts are the causes of seminal losses. We have no evidence that these pathological conditions exist except, it may be, in very rare instances; and, if so, the application of nitrate of silver to the prostatic mucous membrane in every case of nocturnal emission must be unnecessary; and in spite of its alleged harmlessness, I consider it to be a dangerous treatment. I have known two persons die from the effects of the *porte-caustique*, and I have seen others suffer severely from its employment. This may not be the usual result; but I do say that the application of nitrate of silver to the urethra, whether in stick or in strong solution, is at least a very sharp remedy, and will often produce violent inflammation, and sometimes lay the foundation of a stricture or of a chronic irritation of the bladder.

If, then, caustic be applied on an incorrect surmise as to the condition of, and its effects upon, the prostatic mucous membrane and ejaculatory ducts, it is not only an unnecessary, but, in my opinion, an unsafe method of treatment.—*Gascoyne—British Med. Journal.*

